# Circulatory Immune Cells in Cushing Syndrome: Bystanders or Active Contributors to Atherometabolic Injury? A Study of Adhesion and Activation of Cell Surface Markers

**DOI:** 10.1155/2017/2912763

**Published:** 2017-09-20

**Authors:** Gloria Aranda, Cristina Lopez, Rebeca Fernandez-Ruiz, Yaiza Esteban, Guillermo Garcia-Eguren, Mireia Mora, Irene Halperin, Gregori Casals, Joaquim Enseñat, Felicia A. Hanzu

**Affiliations:** ^1^Group of Endocrine Disorders, IDIBAPS, Barcelona, Spain; ^2^Department of Endocrinology and Nutrition, Hospital Clinic Universitari, Barcelona, Spain; ^3^Cytometry Department, IDIBAPS, Barcelona, Spain; ^4^Centro de Investigación en Red de Diabetes y Enfermedades Metabólicas Asociadas (CIBERDEM), Barcelona, Spain; ^5^University of Barcelona, Barcelona, Spain; ^6^Biochemistry and Molecular Genetics Service, Hospital Clinic Universitari and IDIBAPS and CIBERehd, Barcelona, Spain; ^7^Department of Neurosurgery, Hospital Clinic Universitari, Barcelona, Spain

## Abstract

Glucocorticoids (GC) induce cardiometabolic risk while atherosclerosis is a chronic inflammation involving immunity. GC are immune suppressors, and the adrenocorticotrophic hormone (ACTH) has immune modulator activities. Both may act in atherothrombotic inflammation involving immune cells (IMNC). *Aim*. To investigate adhesion and activation surface cell markers (CDs) of peripheral IMNC in endogenous Cushing syndrome (CS) and the immune modulator role of ACTH. *Material and Methods*. 16 ACTH-dependent CS (ACTH-D), 10 ACTH-independent (ACTH-ID) CS, and 16 healthy controls (C) were included. Leukocytes (Leuc), monocytes (MN), lymphocytes (Lym), and neutrophils (N) were analyzed by flow cytometry for atherosclerosis previously associated with CDs. *Results*. Leuc, N, and MN correlated with CS (*p* < 0.05), WC (*p* < 0.001), WHR (*p* = 0.003), BMI (*p* < 0.001), and hs-CRP (*p* < 0.001). CD14^++^CD16^+^ (*p* = 0.047); CD14^+^CD16^++^ (*p* = 0.053) MN; CD15^+^ (*p* = 0.027); CD15^+^CD16^+^ (*p* = 0.008) N; and NK-Lym (*p* = 0.019) were higher in CS. CD14^+^CD16^++^ MN were higher in ACTH-ID (8.9 ± 3.5%) versus ACTH-D CS (4.2 ± 1.9%) versus C (4.9 ± 2.3%). NK-Lym correlated with c-LDL (*r* = 0.433, *p* = 0.039) and CD15^+^ N with hs-CRP (*r* = 0.446, *p* = 0.037). In multivariate analysis, Leuc, N, and MN depended on BMI (*p* = 0.021), WC (*p* = 0.002), and WHR (*p* = 0.014), while CD15^+^ and CD15^+^CD16^+^ N on hypercortisolism and CS (*p* = 0.035). *Conclusion*. In CS, IMNC present changes in activation and adhesion CDs implicated in atherothrombotic inflammation. ACTH-IDCS presents a particular IMNC phenotype, possibly due to the absence of the immune modulator effect of ACTH.

## 1. Introduction

Chronic hypercortisolism, regardless of its exogenous or endogenous etiology, is marked by an increased cardiometabolic risk pattern consisting of abdominal visceral adiposity, arterial hypertension, insulin resistance/impaired glucose tolerance/type 2 diabetes mellitus (T2D), dyslipidemia, and hypercoagulability, all leading to premature atherosclerosis and increased cardiovascular mortality [1].

What is more is that glucocorticoids (GC) mediate stress response and exert potent immune-suppressive and anti-inflammatory effects. Innate and adaptive immunity response induced by acute or chronic, mainly exogenous, hypercortisolism has been investigated in different experimental settings. Both animal and human studies have shown that various forms of stress are associated with deregulation of the immune function [2, 3]. Furthermore, neuropeptidic components of the hypothalamic-pituitary-adrenal (HPA) axis such as adrenocorticotrophic hormone (ACTH) and B-endorphin possess potent immune-modulatory activities [3, 4].

In Cushing syndrome (CS), the prolonged endogenous exposure to pathologic GC levels induces alterations of the white blood cell count and function with granulocytosis, increased monocytes, and a reduced number of lymphocytes with a decreased CD4/CD8 T ratio and Natural killer (NK) cell activity [5].

It is widely recognized that atherosclerosis is a chronic inflammatory state associated with visceral obesity, T2D, dyslipidemia, and other risk factors [6]. Both innate and adaptive immunity are involved in this inflammatory response [7]; the first steps implicate monocytes while later steps involve CD4, CD8, and NK T lymphocytes as well as recently demonstrated neutrophils [7–11].

Hence, research on the innate and adaptive immunity response both in chronic, mostly exogenous, and in acute experimental-induced hypercortisolism has been performed so far. GC and ACTH may act both independently and together in the atherothrombotic inflammatory pathways.

To date, there have been no reports regarding the adhesion and activation of cell surface markers of peripheral immune cells involved in chronic endogenous hypercortisolism or on the potential immune modulator role of ACTH on these markers.

In the present study, we analyze the immune cell pattern in an endogenous hypercortisolemic state in order to investigate their atherovascular risk phenotype and to evaluate the immune modulator role of ACTH on this pattern. For this purpose, peripheral immune cells—monocytes, lymphocytes, and neutrophils from patients with ACTH-dependent CS (ACTH-D CS) and ACTH-independent CS (ACTH-ID CS)—were analyzed by flow cytometry and compared with those in healthy controls.

## 2. Material and Methods

### 2.1. Subjects

A cross-sectional study was performed in endogenous CS patients referred by diagnosis to the Endocrinology and Nutrition Department of the Hospital Clínic of Barcelona between January 2012 and January 2016. All included subjects were women of Caucasian origin. The study was approved by the ethical committee of the Hospital Clinic of Barcelona, and all participants signed an informed consent. The diagnosis of endogenous CS was established according to the guidelines of both the European Society of Endocrinology (ESE) and the Endocrine Society [12] and shown by repeated elevated levels of urinary free cortisol, loss of circadian rhythm (elevated free night salivary cortisol), and the lack of suppression of cortisol secretion after dexamethasone. Localization of the cortisol-secreting tumour was determined by the ACTH level, cortisol suppression test with 8 mg dexamethasone, imaging tests, and inferior petrosal sinus sampling (IPSS) when necessary [12]. The subjects included were as follows:

1. Sixteen patients with pituitary ACTH-dependent CS with age at diagnosis 44.1 ± 13.5 years.
2. Ten patients with ACTH-independent CS with age at diagnosis 43.8 ± 9.8 years.
3. Sixteen healthy subjects (age 43.6 ± 10.3 years) recruited among the relatives of the CS patients and volunteers. None presented signs or symptoms of hypercortisolism, a history of adrenal incidentaloma, or severe and/or chronic illness. None was taking exogenous glucocorticoids or drugs that could interfere with the HPA axis. Controls were matched by age with CS patients.

### 2.2. Anthropometric and Clinical Parameters

All subjects were examined after a 10 h overnight fast. Weight, height, BMI, and waist and hip circumference were measured. Fasting blood samples were taken and either stored at −80°C or directly processed for immune cell phenotyping.

Body weight was measured in light clothing and without shoes to the nearest 0.1 kg. Height was measured to the nearest half centimeter. BMI was calculated as weight in kilograms divided by height in square meters (kg/m^2^). Waist circumference (WC) was measured at minimal inspiration to the nearest 0.1 cm, midway between the last rib and the iliac crest. Hip circumference was measured at the widest of the buttocks and then calculated the waist/hip ratio (WHR).

### 2.3. Laboratory Parameters

Blood samples were collected at 8 a.m., after overnight fasting. Main biochemical parameters were measured in serum with standard methods in the core laboratory of our hospital. High-sensitivity C-reactive protein (hs-CRP) was determined using an immunonephelometric method (Boehring Nephelometer analyzer; Dade Boehring, Marburg, Germany). Plasma glucose, total and HDL cholesterol, and triglycerides were measured using ADVIA 2400 (Siemens Healthcare Diagnostics, Tarrytown, NY, USA), and HbA1C was measured using high-performance liquid chromatography (A. Menarini Diagnostics, Firenze, Italy).

Insulin was determined in duplicate by an ELISA kit (Mercodia AB, Uppsala, Sweden) following the manufacturer instructions. The intra- and interassay CVs were lower than 4% and the assay sensitivity was 1 mU/l. Insulin resistance was calculated according with the homeostasis model assessment (HOMA-IR): insulin resistance = fasting plasma insulin (*μ*U/ml) × fasting plasma glucose (mmol/l)/22.5.

The hormonal analysis was performed in the hormonal laboratory of our centre, applying the standard procedures as follows: ACTH was measured by IRMA (ACTH-IRMA; DiaSorin, Saluggia, Italy); salivary cortisol was analyzed with a specifically validated competitive immunoassay (Salimetrics LLC, State College, Pennsylvania, USA). Serum cortisol was measured using a chemiluminometric immunoassay ran on ADVIA Centaur XP Immunochemistry analyzer (Siemens Healthcare Diagnostics, Tarrytown, NY, USA). The concentration of 24 h urine cortisol was analyzed by chemiluminescence immunoassay (Liaison; DiaSorin, Saluggia, Italy) and confirmed by gas chromatography mass spectrometry.

### 2.4. Immune Cell Phenotyping

Fresh whole blood was obtained after overnight fasting. White cell subpopulations were analyzed in a 100 *μ*l whole-blood assay following standard protocols [13, 14]. Fluorochrome-conjugated monoclonal antibodies to cell surface markers of adhesion and activity were purchased from BD Bioscience (San Diego, CA, USA) and used in different combinations in order to analyze lymphocytes (CD16-PeCy7, CD25-Brilliant Blue 605, CD3-FITC, CD4-PECy5′5, CD19-APC, CD11b-AF700, and CD8-APCCy7) or monocytes/granulocytes (CD16-PeCy7, CD14-Pacific Blue, CD11b-AF700, and CD15-PE). Samples were evaluated as follows by blood flow cytometry using an LSRFortessa™ SORP with the FACSDiva Software v6.1.3 (both Becton Dickinson).

**Figure 1:**
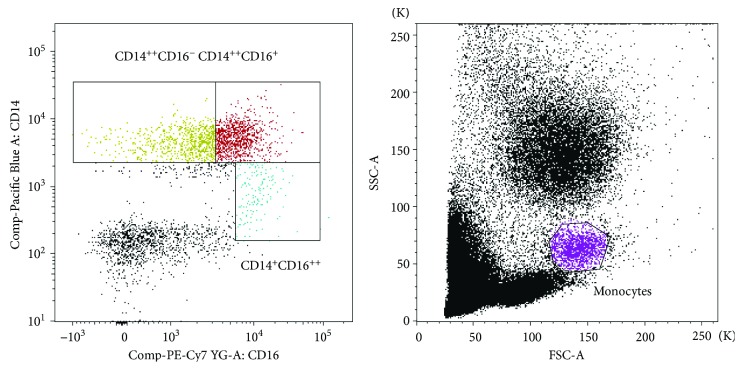
Flow cytometry analysis of activation and adhesion cell marker on monocytes in peripheral blood. Monocytes were gated based on morphological characteristics using their forward (FSC) and side scatter (SSC) properties. CD14^++^CD16^−^, CD14^++^CD16^+^, and CD14^+^CD16^++^ populations were determined and thereafter the % CD11b in each of these populations as well as % of total CD14^+^ and CD14^+^CD11b^+^.

Counting beads were employed for absolute number evaluation. The sizes of specific immune cell subsets were expressed as a percentage of the total white blood cell count. Monocytes were first gated based on morphological characteristics using their forward (FSC) and side scatter (SSC) properties. Next, we identified the three main monocyte subpopulations in a dot plot of CD14 versus CD16: CD14^++^CD16^−^, CD14^++^CD16^+^, and CD14^+^CD16^++^. In each of these populations, the percentages of CD11b^+^ cells were calculated. The percentage of total CD14^+^ cells and CD14^+^CD11b^+^ cells was determined too (Figure 1).

T lymphocytes were defined as CD3^+^ cells, and we distinguished between CD3^+^CD4^+^ and CD3^+^CD8^+^ cells. Expression of CD25 was analyzed on CD3^+^CD4^+^ T cell subpopulation taking CD3^+^CD8^+^ T cells as negative control for CD25 expression. Next, we established the CD3^−^CD19^+^ (B cells) and CD3^−^CD19^−^ subpopulations in the invert gate of CD3^+^ cells (not CD3^+^ events). In order to define NK cell population, we gated CD4^−^CD8^−^ cells and combined them with CD3^−^CD19^−^ cells. Then, we show this population in a CD19 versus CD14 dot plot and select those events, which were also CD14. Finally, NK cells were defined as CD3^−^CD19^−^CD14^−^CD16^+^ events, where we determined the percentage of NK CD11b^+^ cells.

Granulocytes were gated according to their morphological characteristics and then analyzed for the expression of CD15, CD16, and CD11b. We calculated the percentage of CD15^+^, CD16^+^ total granulocytes, the percentage of CD15^+^CD11b^+^, CD16^+^CD11b^+^, CD15^+^CD16^+^ double positive cells, and also CD15^+^CD16^+^CD11b^+^ triple positive cells.

### 2.5. Statistical Analysis

Due to our small sample size and the abnormal distribution of most of the variables with the Kolmogorov-Smirnov test, we underwent nonparametric tests to analyze the sample. Quantitative data are expressed as mean and SD, and qualitative data are expressed as percentages. Comparisons between the CS group and controls were performed using nonparametric tests and between ACTH-ID and ACTH-D CS and controls using ANOVA test followed by Bonferroni analysis when assuming equal variances or Games-Howell when assuming different variances. Correlations between anthropometric parameters, laboratory and hormonal parameters, and phenotyping parameters (immune cells) across the study population were assessed using the Pearson method. Multivariate analysis was performed to analyze the immune cell dependence from variables that show significant association in univariate analyses (BMI, WHR, WC, hs-CRP, 24-UFC, and ACTH). All analyses were performed using SPSS version 22 (SPSS, Chicago, IL, USA), and the level of significance was established at the two-sided 5% level.

## 3. Results

Clinical characteristics of patients and controls are presented in Table 1. There were no differences in age and BMI among patients. Patients with CS presented with typical cardiometabolic comorbidities induced by hypercortisolism like insulin-resistance, T2D, hypertension, and obesity. The entire CS group presented differences in WC, WHR, total and LDL-c cholesterol, and hs-CRP in relation with controls.

### 3.1. Immune Cell Phenotyping

Total leukocytes, neutrophils, and monocytes were increased in all patients with CS, both in the ACTH-dependent and ACTH-independent forms, while there were no differences in lymphocyte counts (Table 1).

#### 3.1.1. Monocytes

Controls presented an increased percentage of classical CD14^++^CD16^−^ monocytes (*p* = 0.082), whereas CD14^++^CD16^+^ intermediate monocytes (*p* = 0.047) were higher in the CS group, both in the ACTH-dependent and ACTH-independent forms, with the highest atypical CD14^+^CD16^++^ monocytes in patients with ACTH-independent CS (*p* = 0.019). Activated CD11b^+^ immune cell phenotypes were increased in all analyzed immune cell subtypes in patients with ACTH-independent CS, but without reaching a statistically significant difference (Tables 2 and 3).

#### 3.1.2. Neutrophils

We observed a higher percentage of CD15^+^ (*p* = 0.027) and CD15^+^CD16^+^ (*p* = 0.008) and tendency in CD15^+^CD16^+^11b^+^ neutrophils in the CS group than in the control, whereas CD16^+^11b neutrophils showed no differences (Tables 2 and 3).

#### 3.1.3. Lymphocytes

We observed no differences in lymphocyte subtypes, except for the prevalence of *NK* (*p* = 0.019) *lymphocytes* that were higher in the CS group (Tables 2 and 3).

### 3.2. Correlations between Immune Cells and Clinical and Analytical Parameters

**Figure 2:**
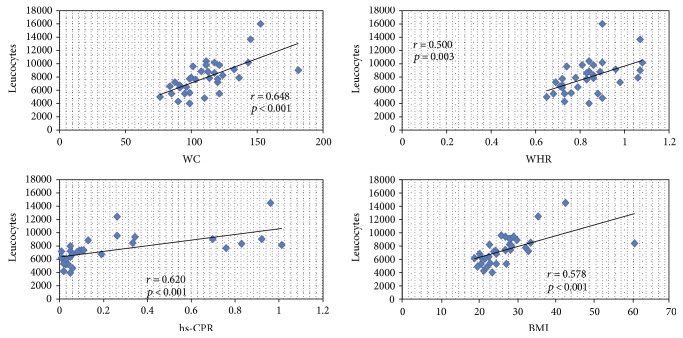
Correlations between leukocytes and clinical and analytical parameters. BMI: body mass index; hs-CRP: high-sensitivity C-reactive protein; WC: waist circumference; WHR: waist/hip ratio. Total leukocyte, monocyte, and neutrophil number correlates with waist circumference (*p* < 0.001), waist/hip ratio (*p* = 0.003), BMI (*p* < 0.001), and hs-CRP (*p* < 0.001).

A positive correlation was observed between leukocytes and waist circumference (*p* < 0.001), WHR (*p* = 0.003), BMI (*p* < 0.001), and hs-CRP (*p* < 0.001). The same correlation was observed for neutrophils and monocytes. NK-lymphocyte positive correlate with c-LDL (*r* = 0.433, *p* = 0.039) and CD15^+^ neutrophils with hs-CRP (*r* = 0.446, *p* = 0.037) (Figure 2). In multivariate analysis, we observed that leukocyte, neutrophil, and monocyte numbers depend on BMI [*p* = 0.021, OR: −1.21 (IC: −3.03 to −0.15)], WC [*p* = 0.002, OR: 3.15 (IC: 1.21 to 5.03)], and WHR [*p* = 0.014, OR: −1.2 (IC: −2.98 to −0.18)]. When we analyzed the phenotyped immune cells, we observed that CD15^+^ neutrophil and CD15^+^CD16^+^ neutrophil depend on the hypercortisolism present only in CS [*p* = 0.035, OR: −1.57 (IC: −5.21 to 1.13)].

## 4. Discussion

The HPA axis modulates in a circadian fashion the endogenous inflammatory and stress response pathways with glucocorticoids as final tissue effectors. Immune cells play an important role in macrovascular inflammation associated to systemic atherosclerosis perpetuating the inflammatory circulatory environment and the chronic and acute injury of the activated endothelium [7–11]. Observational studies have reported that traditional cardiometabolic risk factors as insulin-resistant visceral obesity, T2D, and dyslipidemia associate with changes in the peripheral immune cell pattern [13–22].

Glucocorticoid excess is marked by an increased cardiometabolic risk pattern consisting of visceral adiposity, arterial hypertension, insulin resistance/impaired glucose tolerance/T2D, dyslipidemia, and hypercoagulability, leading to premature atherosclerosis and increased cardiovascular mortality [1]. Interestingly, overphysiologic chronic levels of endogenous or exogenous GC induce immune suppression and increased cardiovascular morbidity and mortality suggesting a possible dual effect of GC on both metabolic and immune pathways in the atherosclerotic cascade. Moreover, the role of the upper HPA axis elements, such as ACTH as a potential immune modulator in atherosclerotic inflammation, has not yet been evaluated.

Thus, we analyzed the potential effects of GC and ACTH on the peripheral atherovascular immune cell surface markers of patients with endogenous ACTH-dependent and ACTH-independent CS and of healthy controls. We studied the cell surface adhesion and activation markers previously associated with chronic atherosclerosis inflammation in circulatory monocytes, lymphocytes, and neutrophils. The present study has its limitations: since endogenous CS is a rare disease, our study population is small and we have included only women, which may have diminished the statistical power of our results.

Evidence of immune cell alterations in endogenous CS, other than the known increased risk of infections observed in clinical settings, has been accumulated over several decades. Meanwhile, major knowledge about GC immune actions has been proven by various studies of innate and adaptive immunity response in chronic, mostly exogenous or acute experimental-induced hypercortisolism.

Elevated white blood cell count and neutrophilia associated with CS were described early in the 1940s [23]. According to epidemiological and experimental data, leukocytosis is an independent risk factor and predictor of future cardiovascular events [24]. In the late 1990s, Kronfol et al. reported a relative decrease in the percentage of CD4^+^ and a relative increase in that of CD8^+^ T cells, together with reduced CD4/CD8 ratio and NK T cell activity, in patients with CS as compared to matched controls [3]. Later on, Masera et al. observed both *in vivo* and *in vitro* that the spontaneous and cortisol-dependent percentage inhibition NK activity was different in ACTH-dependent versus ACTH-independent CS. In pituitary-dependent CS, plasma ACTH correlated positively with mean levels of spontaneous NK activity and negatively with cortisol-dependent percentage inhibition. In adrenal-dependent CS, a negative correlation was observed between spontaneous NK activity and urinary free cortisol. These findings, suggested an immune modulator role of pituitary pro-opiomelanocortin-derived peptides, like ACTH, that effectively counterbalance, at least partially, GC immunosuppression [25].

In our series, the correlation analysis between phenotype markers and immune cells revealed that the CS patients presented leukocytosis with neutrophilia and monocytosis that positively correlated with WC, WHR, BMI, and hs-CRP, an association that persists except for hs-CRP in multivariate analysis.

Detailed analysis of our results points out compelling changes in adhesion and activation markers of vascular inflammation in circulatory immune cells in CS.

Among lymphocytes, NK lymphocytes were significantly increased in CS and correlated with LDL-cholesterol; unlike Kronfold et al., we observed that no difference between ACTH and ACTH-ID CS NK cells has been shown to react toward lipid antigens presented by CD1 molecules on antigen-presenting cells and, once activated, to produce proinflammatory cytokines and promote atherosclerosis [8, 11, 26].

Interestingly, besides an increase in neutrophil number, as seen in experimental data from hypercortisolemic conditions [27, 28], the neutrophils of all CS patients showed a tendency to express activation markers like FC receptor CD16 and tetrasaccharide adhesion marker CD15, an association that persists in multiregression analysis. CD15^+^ neutrophils associated with the higher systemic inflammation observed in the CS patients. Just a slight tendency for an increased expression of integrin adhesion molecule CD11b was observed in ACTH-independent CS.

Sustained clinical evidence supports the association of neutrophilia with morbid obesity and metabolic syndrome, as well as an increased morning cortisol in obese subjects [29, 30].

The importance of neutrophils in the onset and progression of atherosclerosis has been recently reported, probably due to their rapid turnover in atherosclerotic plaque and also the lack of adequate detection methods. Neutrophils have been reported as associated with acute coronary events and as independent predictors of multiple stenosis in chronic stable angina. In patients with coronary artery disease undergoing stent implantation, ACTH and cortisol patterns modulate neutrophil activation. Neutrophils adhere to the plated-activated endothelium and trigger recruitment of inflammatory monocytes and T lymphocytes. Thereafter, they activate proinflammatory cytokine secretion by macrophages and interact with T cell subsets. Moreover, in advanced atherosclerotic lesions, they favor plaque rupture by secretion of matrix degradation factors [9, 10, 31, 32].

Strikingly, in our series, the analysis of the circulating monocyte patterns revealed the presence of higher levels of intermediate CD14^++^CD16^+^ and nonclassical CD14^+^CD16^++^ cardiovascular risk monocyte phenotypes in CS patients; among them, the highest levels of atypical monocytes were observed in ACTH-independent CS patients.

Monocytes have been divided into 3 major subsets according to the expression intensity of lipopolysaccharide (LPS) receptor CD14 and FC receptor CD16 [33]. Classical CD14^++^CD16^−^ monocytes are professional phagocytes that ingest native low-density lipoprotein, (LDL), generate reactive oxygen species (ROS) that secrete cytokines in response to LPS [15], and activate CD4 T cells. In contrast, intermediate CD14^++^CD16^+^ and nonclassical or atypical CD14^+^CD16^++^ monocytes do not generate ROS and are weak phagocytes taking up preferentially oxidized LDL, but they are able to control the endothelium through their capability to crawl on the endothelium-blood interface. Intermediate CD14^++^CD16^+^ monocytes increase in inflammatory and autoimmune conditions and seem to inhibit CD4^+^ T cell proliferation induced by other monocyte subsets and enhance CD4^+^ T regulatory IL-10 expression. Nonclassical CD 14^+^CD16^++^ monocytes are potent proinflammatory cells related to cardiovascular disease and substantially secrete inflammatory cytokines, like tumour necrosis factor-*α*, interleukin-1*β*, and CCL3 [15–18]. CD16^+^ monocytes have been generally associated with traditional cardiovascular risk factors like insulin-resistant obesity, systemic low-grade inflammation diabetes, and dyslipidemia and also with atherosclerosis and acute coronary events. CD16^+^ monocytes also actively contribute to infarct healing, remodeling, and angiogenesis. Nevertheless, the specific role of monocyte subsets is still at debate [14, 19–22]. Moreover, no evidence is available on monocyte CD14CD16 profiles in relation to the HPA axis.

Our results, the first focused on endogenous exposure to GC, were similar to those of Liu et al., who showed increased intermediate CD14^+^CD16^+^ monocytes after treatment in patients with autoimmune disease [18]; moreover, GC treatment directly affected CD16 expression. Nevertheless, our data in CS patients, an endogenous chronic state of hypercortisolism, differ from other previous findings after GC administration, either acute or subacute, in controls [34] and patients with inflammatory disorders [35–37] or chronic low doses in patients with chronic inflammatory diseases [38].

## 5. Conclusion

We have analyzed for the first time adhesion and activation of cell surface markers of peripheral immune cells in a chronic pathologic endogenous hypercortisolemic state. Here, we report that chronic exposure to high, pathological levels of GC reaching an overt CS state independently associates with increased levels of CD15^+^CD16^+^ and CD15^+^ neutrophils and correlates with intermediate CD14^++^CD16^+^ and nonclassical CD14^+^CD16^++^ monocyte induction, due to the overall effect of GC-induced metabolic features. Possible exceptions may be the activation of neutrophils which may have a dual dependency from both metabolic changes and hypercortisolism pathways per se and also the unique pattern that characterizes monocyte subset distribution in ACTH-independent CS. Stronger monocyte changes in ACTH-independent hypercortisolism may be due to the absence of the immune-modulatory effect of ACTH. This study opens new research standpoints concerning the crosstalk between adrenal axis and leucocyte subsets in insulin-resistant obesity and their role in metabolic-driven atherovascular disease.

## Figures and Tables

**Table 1:** Characteristics of the study population.

Parameters	ACTH-dependent CS (*n* = 16)	ACTH-independent CS (*n* = 10)	Control (*n* = 16)	*p* value
Age (years)	44.1 ± 13.5	43.8 ± 9.8	43.9 ± 10.3	0.869
Leukocytes (mm^3^)	9460.0 ± 2429.2	7508.7 ± 1329.8	5842.0 ± 1279.2	**<0.001** ^∗∗^
Neutrophils (mm^3^)	6562.5 ± 2093.7	5062.5 ± 1329.8	3270.0 ± 960.4	**<0.001** ^∗∗^
Lymphocytes (mm^3^)	2031.2 ± 710.6	1725.0 ± 549.6	1870.0 ± 533.4	0.522
Monocytes (mm^3^)	562.5 ± 224.7	425.0 ± 128.1	330.0 ± 94.8	**0.008** ^∗^
T2D (%)	5 (31)	3 (30)	0 (0)	0.341
HTA (%)	8 (50)	5 (50)	0 (0)	0.145
DLP (%)	3 (19)	3(30)	0 (0)	0.271
Obesity (%)	8 (50)	5 (50)	1 (6)	0.215
BMI (kg/m^2^)	29.4 ± 5.1	29.2 ± 13.1	24.2 ± 4.2	0.339
Waist C (cm)	94.7 ± 14.1	94.2 ± 18.3	77.5 ± 11.7	**0.037** ^∗^
WHR	0.90 ± 0.1	0.89 ± 0.1	0.71 ± 0.1	**0.024** ^∗^
TC (mmol/l)	5.6 ± 0.8	5.5 ± 0.5	4.5 ± 0.5	**0.020** ^∗^
TG (mmol/l)	1.4 ± 0.6	1.4 ± 0.5	0.9 ± 0.6	0.238
LDL-c (mmol/l)	3.4 ± 0.5	3.4 ± 0.6	2.5 ± 0.6	**0.016**
HDL-c (mmol/l)	1.5 ± 0.4	1.5 ± 0.3	1.7 ± 0.6	0.355
Hs-CRP (mg/dl)	0.36 ± 0.36	0.30 ± 0.31	0.02 ± 0.01	**0.038** ^∗^
Glucose (mmol/l)	6.5 ± 2.7	6.1 ± 0.7	5.2 ± 0.6	0.268
HOMA-IR	3.80 ± 2.51	3.69 ± 2.23	2.89 ± 2.91	0.482
HbA1c (DCCT)	6.8 ± 1.9	6.6 ± 0.5	5.4 ± 0.2	0.076
UFC-24hs (mcg/24hs)	269.2 ± 114.8	257.7 ± 118.1	44.8 ± 12.6	**0.029** ^∗^
ACTH (pg/ml)	68.4 ± 73.5	5.6 ± 1.4	33.5 ± 7.3	**0.023** ^∗^

Data are expressed as mean ± standard deviation. Qualitative variables are expressed as percentages. BMI: body mass index; HOMA-IR: homeostasis model assessment for insulin resistance; T2D: type 2 diabetes; HbA1c (DCCT): haemoglobin A1c (diabetes control and complication trial units); Hs-CRP: high-sensitivity C-reactive protein; LDL-c: low-density lipoprotein cholesterol; HDL-c: high-density lipoprotein cholesterol; WHR: waist/hip ratio. ^∗^*p* < 0.05. ^∗∗^*p* < 0.001.

**Table 2:** Cell surface marker phenotyping of immune cells between CS and controls.

Parameters	Controls (*n* = 16)	Cushing syndrome (*n* = 26)	*p* value
*Monocytes*			
CD14^++^CD16^−^ classical (MN)	42.2 ± 32.5	34.2 ± 30.1	0.082
CD14^++^CD16^−^11b^+^ (%)	80.8 ± 19.6	72.4 ± 32.4	0.227
CD14^++^CD16+ intermediate (MN)	28.7 ± 26.1	36.3 ± 25.1	**0.047** ^∗^
CD14^++^CD16^+^11b^+^ (%)	48.1 ± 48.7	52.4 ± 44.8	0.432
CD14^+^CD16^++^ nonclassical (MN)	5.8 ± 3.8	7.1 ± 2.3	**0.053** ^∗^
CD14^+^CD16^++^11b^+^ (%)	35.4 ± 41.1	38.4 ± 42.8	0.864
*Neutrophils*			
CD15^+^ (% N)	47.1 ± 13.9	65.8 ± 24.6	**0.027** ^∗^
CD15^+^CD16^+^ (%)	44.3 ± 12.7	66.6 ± 25.2	**0.008** ^∗^
CD15^+^CD16^+^11b (%)	69.2 ± 29.1	80.6 ± 26.6	0.098
CD16^+^11b (% N)	94.2 ± 4.2	90.0 ± 22.7	0.460
*Lymphocytes*			
CD3^+^CD8^+^ (% L)	26.8 ± 8.0	30.1 ± 11.9	0.435
CD3^+^CD4^+^ (% L)	54.2 ± 6.6	47.6 ± 18.6	0.207
CD3^+^CD4^+^CD25^+^ (%)	22.7 ± 11.1	20.7 ± 18.8	0.746
NK (% L)	18.4 ± 18.5	34.7 ± 32.2	**0.019** ^∗^
NKCD11b^+^ (%)	37.5 ± 44.4	35.1 ± 36.0	0.889

Data are expressed as % ± standard deviation. MN: monocytes; N: neutrophils; L: lymphocytes. ^∗^*p* < 0.05.

**Table 3:** Cell surface marker phenotyping of immune cells.

Parameters	ACTH-dependent CS (*n* = 16)	ACTH-independent CS (*n* = 10)	Controls (*n* = 16)	*p* value
*Monocytes*				
CD14^++^CD16− classical (% MN)	32.7 ± 29.2	36.4 ± 33.8	42.2 ± 32.5	0.805
CD14^++^CD16^−^11b^+^ (%)	63.4 ± 37.5	86.6 ± 15.5	80.8 ± 19.6	0.195
CD14^++^CD16^+^ intermediate (% MN)	37.6 ± 25.1	34.2 ± 27.0	28.7 ± 26.2	0.748
CD14^++^CD16^+^11b^+^ (%)	47.7 ± 43.7	59.8 ± 49.1	48.1 ± 48.7	0.845
CD14^+^CD16^++^ Nonclassical (% MN)	4.9 ± 1.9	8.9 ± 3.5	5.8 ± 3.8	**0.019** ^∗^
CD14^+^CD16^++^11b^+^ (%)	29.1 ± 38.7	52.9 ± 48.0	35.4 ± 41.1	0.608
*Neutrophils*				
CD15^+^ (% N)	65.5 ± 27.5	66.3 ± 21.2	47.1 ± 13.9	0.205
CD15^+^CD16^+^ (%)	64.4 ± 28.0	70.2 ± 21.6	44.3 ± 12.7	0.103
CD15^+^CD16^+^ 11b (%)	74.2 ± 30.7	90.5 ± 15.3	69.2 ± 29.1	0.313
CD16^+^11b (% N)	85.9 ± 28.8	96.4 ± 1.5	94.2 ± 4.2	0.495
*Lymphocytes*				
CD3^+^CD8^+^ (% L)	27.9 ± 14.1	33.2 ± 8.0	26.8 ± 8.1	0.496
CD3^+^CD4^+^ (%L)	43.6 ± 21.6	53.4 ± 12.6	54.2 ± 6.6	0.300
CD3^+^CD4^+^CD25^+^ (%)	18.7 ± 16.7	23.7 ± 22.7	22.7 ± 11.1	0.813
NK (% L)	30.8 ± 31.8	40.3 ± 34.4	18.4 ± 18.5	0.359
NK CD11b^+^ (%)	26.4 ± 31.2	47.6 ± 40.9	37.5 ± 44.4	0.541

Data are expressed as % ± standard deviation. MN: monocytes; N: neutrophils; L: lymphocytes. ^∗^*p* < 0.05.
